# Vitamin A deficiency in the MENA region: a 30-year analysis (1990–2019)

**DOI:** 10.3389/fnut.2024.1413617

**Published:** 2024-06-06

**Authors:** Saeid Safiri, Seyed Ehsan Mousavi, Seyed Aria Nejadghaderi, Kimia Motlagh Asghari, Nahid Karamzad, Mark J. M. Sullman, Ali-Asghar Kolahi, Morteza Abdollahi

**Affiliations:** ^1^Social Determinants of Health Research Center, Department of Community Medicine, Faculty of Medicine, Tabriz University of Medical Sciences, Tabriz, Iran; ^2^Clinical Research Development Unit of Tabriz Valiasr Hospital, Tabriz University of Medical Sciences, Tabriz, Iran; ^3^Neurosciences Research Center, Aging Research Institute, Tabriz University of Medical Sciences, Tabriz, Iran; ^4^HIV/STI Surveillance Research Center, WHO Collaborating Center for HIV Surveillance, Institute for Futures Studies in Health, Kerman University of Medical Sciences, Kerman, Iran; ^5^Systematic Review and Meta-analysis Expert Group (SRMEG), Universal Scientific Education and Research Network (USERN), Tehran, Iran; ^6^Department of Persian Medicine, School of Traditional Medicine, Tabriz University of Medical Sciences, Tabriz, Iran; ^7^Nutrition Research Center, Department of Biochemistry and Diet Therapy, School of Nutrition and Food Sciences, Tabriz University of Medical Sciences, Tabriz, Iran; ^8^Department of Life and Health Sciences, University of Nicosia, Nicosia, Cyprus; ^9^Department of Social Sciences, University of Nicosia, Nicosia, Cyprus; ^10^Social Determinants of Health Research Center, Shahid Beheshti University of Medical Sciences, Tehran, Iran

**Keywords:** vitamin A deficiency, Middle East and North Africa, year lived with disability, prevalence, Global Burden of Disease

## Abstract

**Background:**

Vitamin A deficiency (VAD) is a common micronutrient deficiency that imposes a substantial burden worldwide. This study examined the burden of VAD from 1990 to 2019 in the Middle East and North Africa (MENA) region by age, sex and sociodemographic index (SDI).

**Methods:**

The data were obtained from the Global Burden of Disease (GBD) 2019 database. We reported the prevalence, incidence, and years lived with disability (YLDs) that were attributable to VAD for the MENA region, along with its constituent countries.

**Results:**

In 2019, the MENA region had 30.6 million prevalent cases of VAD, with an age-standardized prevalence rate of 5249.9 per 100,000 population. In addition, VAD was responsible for 62.2 thousand YLDs, with an age-standardized YLD rate of 10.2 per 100,000. The age-standardized prevalence [50.3% (−55.9 to −44.7)] and YLD [−49.3% (−55.3 to −43.1)] rates of VAD have significantly decreased since 1990. In 2019, the MENA region’s VAD-attributable YLD rate was below the global average for males and females across all age groups. Additionally, SDI was negatively associated the age-standardized YLD rate of VAD.

**Conclusion:**

This study underscores the necessity of frequently updating health data and developing guidelines and regulations to prevent, detect early, and effectively treat VAD in the MENA countries.

## Introduction

Vitamin A deficiency (VAD) is one of the most common types of micronutrient deficiencies, and in 2019 it had a worldwide age-standardized point prevalence of 6955.6 per 100,000 (1). VAD is related to several different disorders and conditions, such as xerophthalmy and blindness (2), tuberculosis (3), coronavirus disease 2019 (4) and anemia (5). Children under the age of five, as well as pregnant and lactating women, are particularly susceptible to VAD (6). Furthermore, VAD is more prevalent in less developed regions and countries (7).

Hunger and micronutrient deficiencies were widespread public health concerns in the Middle East and North Africa (MENA) (8). The prevalence of VAD in the region has decreased since the United Nations and World Health Organization implemented supplementation programs (9, 10). However, in 2019, the age-standardized incidence rate of VAD was significantly higher in the MENA region than it was globally (5249.9 vs. 6955.7 per 100,000 population) (7). In addition, in 2019 VAD accounted for a large number of disability-adjusted life-years (DALYs) in MENA, despite a remarkable decrease in its incidence over the last three decades (7).

Prior publications have studied the burden of nutritional and micronutrient deficiencies at the global or regional levels (1, 11) and in specific countries (12). Earlier studies have documented the impact of VAD on various health conditions, such as vision loss (2). Furthermore, although the incidence and DALYs associated with VAD have been reported globally from 1990 to 2019, the prevalence data, which is important for resource allocation and policy-making, was not reported (7). Furthermore, the previous study did not focus on the burden of VAD in MENA (7). With this in mind, the current research examined the impact of VAD in the MENA region by age, sex and sociodemographic index (SDI) from 1990 to 2019.

## Methods

### Overview

Data on the burden of vitamin A deficiency (VAD) from 1990 to 2019 were sourced from the Global Burden of Disease (GBD) 2019 database. The age-standardized prevalence and YLD rates of VAD were reported for each MENA country and for the region as a whole, categorized by age group, sex and SDI. MENA is composed of: Afghanistan, Algeria, Bahrain, Djibouti, Egypt, Iran, Iraq, Jordan, Kuwait, Lebanon, Libya, Morocco, Oman, Palestine, Qatar, Saudi Arabia, Sudan, The Syrian Arab Republic, Tunisia, Turkey, the United Arab Emirates, and Yemen. The Institute for Health Metrics and Evaluation (IHME) made several refinements to the methodologies and modeling strategies used to produce the GBD 2019 data, which are outline in previous publications (13, 14). The mortality and morbidity data were obtained from the following websites: https://vizhub.healthdata.org/gbd-compare/ and http://ghdx.healthdata.org/gbd-results-tool.

### Case definition and data sources

In the GBD 2019 study, vitamin A deficiency (VAD) was defined as having a serum retinol level below 0.7 μmol/L. The burden of VAD was assessed by calculating the VAD prevalence, as well as the prevalence of vision loss and blindness that was caused by VAD, which were defined as corneal ulceration and scarring (13).

The IHME used data from a number of sources to model the VAD prevalence, including the WHO Vitamin and Mineral Nutrition Information System, health surveys (e.g., Demographic and Health Surveys –[DHS] and Multiple Indicator Cluster Surveys – [MICS]), as well as other research that was identified during a literature review undertaken by IHME. The literature review conducted for GBD 2013 was utilized for GBD 2019, with following exclusion criteria: studies without population-based method, studies that did not report necessary epidemiological parameters, review studies, case series, and self-reported cases. The UNICEF Children’s Database, as well as the MICS and DHS surveys, were utilized to produce the vitamin A supplement model, while data obtained from the WHO Vitamin and Mineral Information System was utilized to model vision loss. The data utilized for the estimation process are accessible from: https://ghdx.healthdata.org/gbd-2019/data-input-sources.

Previous research was omitted if it met any of the following criteria: (a) they were not sourced from the general population (e.g., studies conducted in hospitals or clinics), (b) they did not report primary epidemiological data, or (c) were reviews, case series, or case reports (13).

### Data processing and disease modeling

The estimation process used in GBD 2019 was similar to that undertaken in GBD 2017, but several improvements were made. The modeling strategy contained three steps, which began with a vitamin A supplementation coverage model. The estimates of vitamin A supplementation were utilized as country-level covariates to inform prevalence estimates of the overall level of vitamin A deficiency, which were then utilized as country-level covariates to model the prevalence of vision loss as a result of VAD. The disparity between the overall prevalence of VAD and the prevalence of vision loss caused by VAD was thought to encompass those individuals who were asymptomatic.

In order to include the most information possible, vitamin A supplementation was modeled first. This model defined cases as the proportion of individuals in one location who had received one or more doses of vitamin A in the last six-months. Unfortunately, most health surveys did not report enough data to use the normal criterion, which is two or more vitamin A doses in the last year for those younger than 5 years old. Following this, Spatiotemporal Gaussian process regression (ST-GPR) was utilized to ensure a more accurate supplementation model across time, since supplementary programs were implemented in the late 1990s.

In earlier GBD iterations, vitamin A supplementation was estimated as an all-age and combined sex indicator, with the case definition being the proportion of those aged 6–59 months that had been given one or more doses of vitamin A during the last 6 months. In order to encapsulate the impact of the supplementation programs on the prevalence of VAD in each age group and sex, in GBD 2019 the supplementation of vitamin A was modeled as an age and sex indicator in order, to reduce the reliance on children aged 6–59 months old.

The second step modeled the prevalence of VAD according to age and sex. In GBD 2019, Meta-Regression using the Bayesian, Regularized, Trimmed method (MR-BRT) was used to develop a sex ratio model and DisMod MR 2.1 was used to produce a separate age model, and these were then utilized to separate the data by sex and into the different age groups prior to modeling. Following this, the data were then modeled using ST-GPR, due to its enhanced ability to model trends over time.

In the third stage, DisMod was used to model the vision loss that was attributable to VAD using a single-parameter meta-regression with the prevalence of VAD as a location-level covariate. The WHO Vitamin and Mineral Information System definition of corneal scar was used as the case definition for estimating the prevalence of vision loss caused by VAD. Prior to DisMod modeling, the sex ratio for vision loss attributable to VAD was modeled using MR-BRT, and the ratio was used to separate the data into sex-specific estimates (13).

### Years of living with a disability

The GBD disability weight survey provided the disability weights (DWs) (Supplementary Table S1) (13). The disability-adjusted life years (DALYs) were estimated by summing the years of life lost as a result of premature mortality and the years of life with a disability (YLDs). As there were no VAD attributable deaths, the YLD and DALY calculations were identical. The severity-specific prevalence estimates were multiplied by their corresponding DW to estimate the YLDs due to VAD.

### Data management and analysis

The 95% uncertainty intervals (UIs) were produced by conducting 1,000 simulations at each stage of the estimation process, incorporating uncertainty from various sources such as source data, error adjustment, and remaining bias estimates (13). The 95% UIs were determined by identifying the 25th and 975th values from the sorted simulations (13). Smoothing Spline models were employed to examine the relationship between the socio-demographic index (SDI) and the burden of VAD (15). The SDI is a development metric that encompasses lagged distributed income *per capita*, average education attainment for individuals aged 15 and older, and fertility rates for women aged 25 and younger. SDI is express on a scale that ranges from 0 to 1 (0 = lowest and 1 = highest level of development) (13). R software (Version 3.5.2) was utilized to create all figures.

## Results

### MENA region

In 2019, there were 30.6 million (95% UI: 28.8–32.4) prevalent cases of VAD, with a point prevalence of 5249.9 (95% UI: 4905.9–5602.5) per 100,000, which represents a 50.3% (95% UI: 44.7–55.9) decrease since 1990 (Table 1 and Supplementary Table S2). There were also 62.2 thousand (95% UI: 41.3–87.4) YLDs in 2019, with an age-standardized rate of 10.2 (95% UI: 6.7–14.3) per 100,000, which represents a 49.3% (95% UI: 43.1–55.3) decrease since 1990 (Table 1 and Supplementary Table S3).

**Table 1:** Prevalence and YLDs due to vitamin A deficiency in the Middle East and North Africa region for both sexes in 2019 and the percentage change in the age-standardized rates during the period 1990–2019.

	Prevalence (95% UI)			YLD (95% UI)		
	Counts (2019)	ASRs (2019)	Pcs in ASRs 1990–2019	Counts (2019)	ASRs (2019)	Pcs in ASRs 1990–2019
North Africa and Middle East	30,635,707 (28,841,772, 32,427,862)	5249.9 (4905.9, 5602.5)	−50.3 (−55.9, −44.7)	62,161 (41,276, 87,376)	10.2 (6.7, 14.3)	−49.3 (−55.3, −43.1)
Afghanistan	9,976,079 (8,681,044, 11,360,871)	26013.8 (22250.9, 30088.7)	−33.2 (−47.1, −15)	17,489 (10,887, 25,833)	28.6 (18, 42.3)	−34.6 (−49.4, −14.1)
Algeria	1,104,506 (936,742, 1,321,371)	2690.4 (2223.7, 3276.3)	−60.9 (−70.6, −44.6)	3,078 (1917, 4,712)	7.3 (4.6, 11.2)	−57.6 (−68.7, −41.7)
Bahrain	20,747 (17,263, 24,956)	1459.9 (1,201, 1773.1)	−67.9 (−79.4, −47.1)	9 (5, 17)	1.2 (0.6, 2.1)	−74.7 (−85.5, −55.7)
Egypt	1,909,494 (1,600,082, 2,270,531)	1925.2 (1590.4, 2320.4)	−62.9 (−72.7, −50)	4,196 (2,572, 6,518)	3.8 (2.4, 6)	−65 (−74.7, −52.9)
Iran	1,105,547 (942,030, 1,334,897)	1344.7 (1118.9, 1651.8)	−90.4 (−93.9, −84.7)	540 (284, 986)	0.8 (0.4, 1.4)	−92.7 (−95.7, −87.3)
Iraq	1,337,629 (1,114,027, 1,602,377)	3209.2 (2619.1, 3937.9)	−62.7 (−73.6, −47.4)	3,353 (2050, 5,099)	7.3 (4.5, 11.1)	−59.6 (−71.3, −45.3)
Jordan	575,229 (487,422, 683,785)	5045.5 (4187.4, 6131.2)	−55.8 (−68.1, −39)	882 (538, 1,335)	7.1 (4.4, 10.7)	−55.9 (−68.5, −38.1)
Kuwait	32,538 (27,566, 39,139)	745.7 (619.9, 912.2)	−25.6 (−38.7, −12.5)	197 (117, 305)	5 (3.1, 7.7)	−26 (−41.1, −9.2)
Lebanon	85,320 (72,497, 101,034)	1682.7 (1399.5, 2043.8)	−77.9 (−86.7, −63.3)	52 (25, 102)	1 (0.5, 2.1)	−84.1 (−91.7, −70.9)
Libya	121,053 (101,641, 144,469)	1829.2 (1515.3, 2213.5)	−52.8 (−66.1, −36.3)	418 (261, 627)	7 (4.4, 10.4)	−46.8 (−60.3, −30.7)
Morocco	2,140,590 (1,798,005, 2,534,391)	6117.3 (5055.5, 7318.7)	−63.2 (−73.2, −48.8)	3,353 (1997, 4,962)	10 (5.9, 14.9)	−64.5 (−74.3, −52)
Oman	59,235 (48,447, 74,110)	1300.8 (1050.1, 1633.5)	−67.6 (−75.7, −57.1)	532 (333, 788)	12.1 (7.7, 18)	−63.3 (−73.1, −52.8)
Palestine	266,716 (222,099, 324,426)	5435.6 (4444.9, 6784.1)	−77.7 (−83.4, −69.4)	582 (357, 876)	9.7 (6, 14.5)	−74.7 (−81.5, −65.2)
Qatar	20,586 (16,451, 26,077)	725.8 (575.1, 927.4)	−81.5 (−89.1, −70.5)	6 (3, 11)	0.4 (0.2, 0.7)	−86.5 (−93.2, −74.6)
Saudi Arabia	66,898 (56,035, 78,991)	190 (156.5, 229)	−95 (−97.2, −91.2)	32 (14, 63)	0.1 (0.1, 0.3)	−96.2 (−98.3, −92.4)
Sudan	3,681,595 (3,088,138, 4,399,984)	9067.5 (7431.2, 11071.5)	−61.1 (−70.2, −48.6)	9,776 (5,951, 14,693)	18.6 (11.3, 27.8)	−64.9 (−73.6, −53.3)
Syrian Arab Republic	529,821 (446,326, 624,305)	3722.4 (3067.3, 4442.2)	−69.6 (−80.5, −53.5)	460 (237, 808)	3.6 (1.9, 6.5)	−75.1 (−84.9, −58.3)
Tunisia	202,042 (173,828, 233,624)	1755 (1,468, 2071.5)	−61.3 (−71.8, −47.9)	490 (305, 744)	4.7 (2.9, 7.1)	−53.1 (−65.6, −39)
Turkey	2,574,870 (2,218,998, 2,951,848)	3184.3 (2713.4, 3724.8)	−79.9 (−86.8, −69.8)	1,060 (541, 1860)	2 (1, 3.5)	−85 (−91.2, −75.6)
United Arab Emirates	66,510 (52,584, 84,088)	729.8 (578.1, 919.2)	−75 (−84.4, −61.2)	23 (11, 41)	0.6 (0.3, 1)	−78.4 (−88.1, −61.9)
Yemen	4,727,579 (3,968,589, 5,544,227)	15059.3 (12423.1, 17887.4)	−46 (−58.9, −30)	15,569 (10,107, 23,336)	36.3 (23.7, 53.6)	−46 (−58.7, −29.8)

### Country level

**Figure 1:**
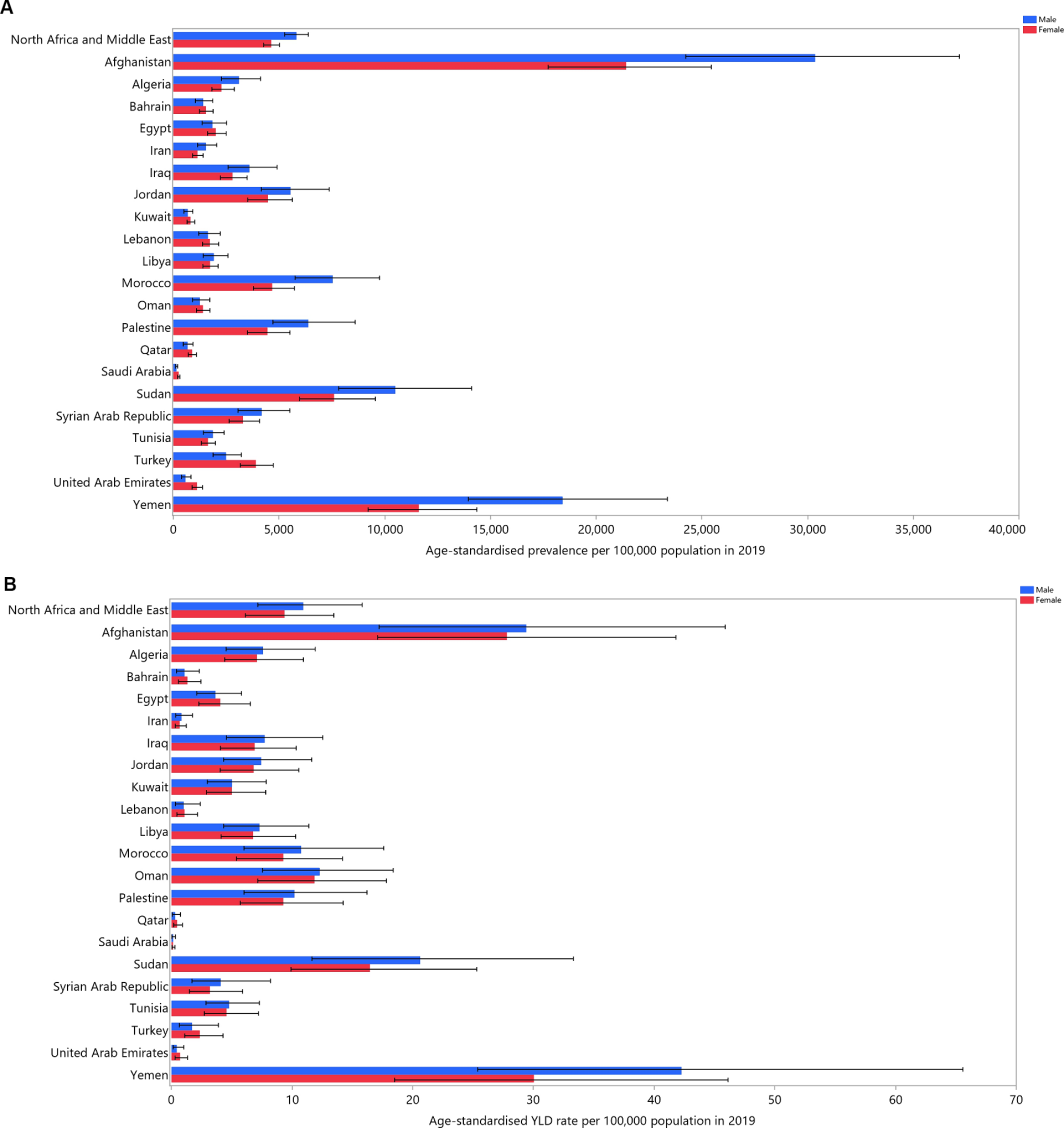
Age-standardized point prevalence **(A)** and YLDs **(B)** for vitamin A deficiency (per 100,000 population) in the Middle East and North Africa region in 2019, by sex and country. YLD = years-lived-with-disability. (Generated from data available from http://ghdx.healthdata.org/gbd-results-tool).

The age-standardized point prevalence of VAD in 2019 showed significant variation, ranging from 190 to 26013.8 cases per 100,000 in the MENA countries. Afghanistan [26013.8 (95% UI: 22250.9–30088.7)], Yemen [15059.3 (95% UI: 12423.1–17887.4)], and Sudan [9067.5 (95%UI: 7431.2–11071.5)] exhibited the highest point prevalence of VAD in 2019. Conversely, Saudi Arabia [190 (95% UI: 156.5–229)], Qatar [725.8 (95% UI: 575.1–927.4)], and the United Arab Emirates [729.8 (95%UI: 578.1–919.2)] were lowest (Supplementary Table S2). The 2019 age-standardized point prevalence of VAD is presented by country and sex in Figure 1A.

At the country level, in 2019 the age-standardized YLD rates varied from 0.4 to 36.3 cases per 100,000. Yemen [36.3 (95% UI: 23.7–53.6)], Afghanistan [28.6 (95% UI: 18–42.3)], and Sudan [18.6 (95% UI: 11.3–27.8)] recorded the highest rates. In contrast, Saudi Arabia [0.1 (95% UI: 0.1–0.3)], Qatar [0.4 (95% UI: 0.2–0.7)], and the United Arab Emirates [0.6 (95% UI: 0.3–1)] were lowest (Supplementary Table S3). The 2019 national age-standardized YLD VAD rates, disaggregated by sex, are illustrated in Figure 1B.

From 1990 to 2019, all MENA countries experienced a substantial reduction in the age-standardized point prevalence of VAD. The most significant reductions were observed in Saudi Arabia [−95% (95% UI: −97.2 to −91.2)], Iran [−90.4% (95% UI: −93.9 to −84.7)], and Qatar [−81.5% (95% UI: −89.1 to −70.5)]. The smallest reductions in the age-standardized point prevalence of VAD were found in Kuwait [−25.6% (95% UI: −38.7 to −12.5)], Afghanistan [−33.2% (95% UI: −47.1 to −15)], and Yemen [−46% (95% UI: −58.9 to −30)] (Supplementary Figure S1 and Supplementary Table S2).

Similarly, the age-standardized YLD rate also decreased across all MENA countries during the same period. The biggest reductions were recorded in Saudi Arabia [−96.2% (95% UI: −98.3 to −92.4)], Iran [−92.7% (95% UI: −95.7 to −87.3)], and Qatar [−86.5% (95% UI −93.2 to −74.6)], while Kuwait [−26% (95% UI: −41.1 to −9.2)], Afghanistan [−34.6% (95% UI: −49.4 to −14.1)], and Yemen [−46% (95% UI: −58.7 to −29.8)] had the smallest (Supplementary Figure S2 and Supplementary Table S3).

### Age and sex patterns

**Figure 2:**
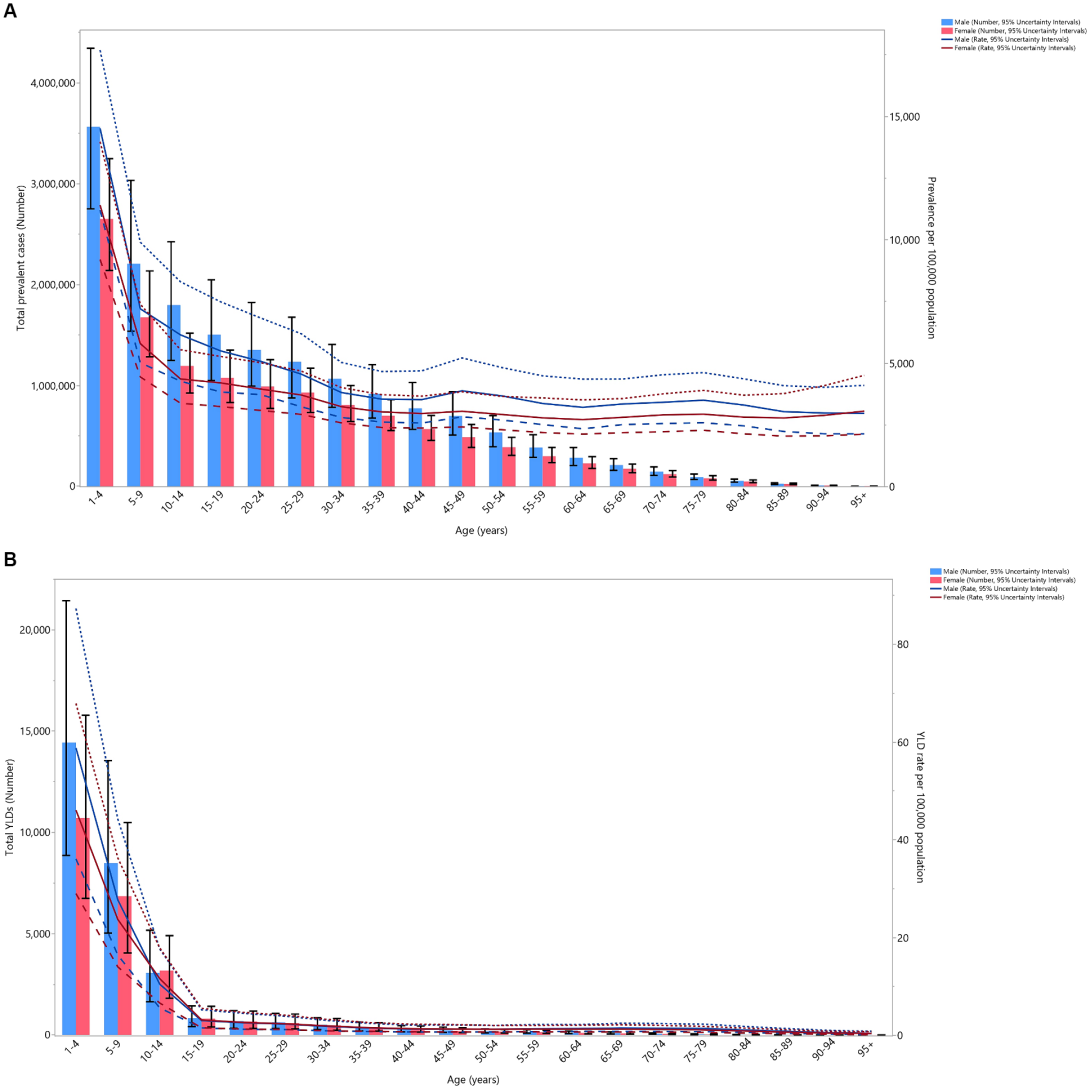
Number of prevalent cases and prevalence **(A)**, and the number of YLDs and the YLD rate **(B)** for vitamin A deficiency (per 100,000 population) in the Middle East and North Africa region, by age and sex in 2019; Dotted and dashed lines indicate 95% upper and lower uncertainty intervals, respectively. YLD = years-lived-with-disability. (Generated from data available from http://ghdx.healthdata.org/gbd-results-tool).

In 2019, both the number of prevalent cases and the point prevalence in the region decreased with age for both sexes. The largest number of prevalent cases and point prevalence were observed among those aged 1–4 years old. The number of prevalent cases dropped sharply for both sexes up to the 10–14 age group, followed by a more gradual decline with advancing age. Similarly, the point prevalence rate for both sexes decreased up to the 30–34 age group, after which it stabilized. There were no significant differences in the number of prevalent cases or the point prevalence of VAD between the two sexes (Figure 2A).

In 2019, the total number of YLDs and the YLD rates decreased dramatically in both sexes up to the 15–19 age group, after which they remained relatively stable with advancing age. No significant sex differences were found in the number of YLDs and the YLD rates (Figure 2B).

**Figure 3:**
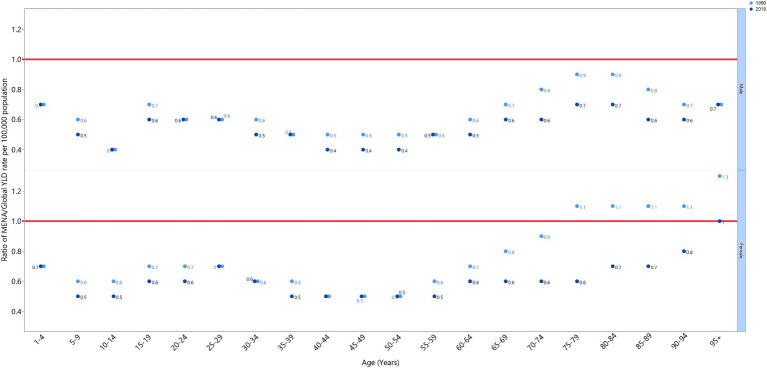
Ratio of the Middle East and North Africa region to the global YLD rate of vitamin A deficiency by age group and sex, 1990–2019. YLD = years-lived-with-disability. (Generated from data available from http://ghdx.healthdata.org/gbd-results-tool).

The VAD-attributed YLD rate in 2019 was lower in the MENA region than it was globally (ratio of MENA/global YLD rate < 1) for both males and females across all age groups, with the exception of females over 95 years old, where the rate was similar to the global rate (MENA/global YLD rate = 1). The MENA/global YLD rate ratios in 1990 were not higher than those found in 2019. The lowest MENA/global YLD rate ratios in 2019 were found in males aged 10–14 and 40–54, and females aged 5–14 and 35–59 (Figure 3).

### Relationship with the socio-demographic index (SDI)

**Figure 4:**
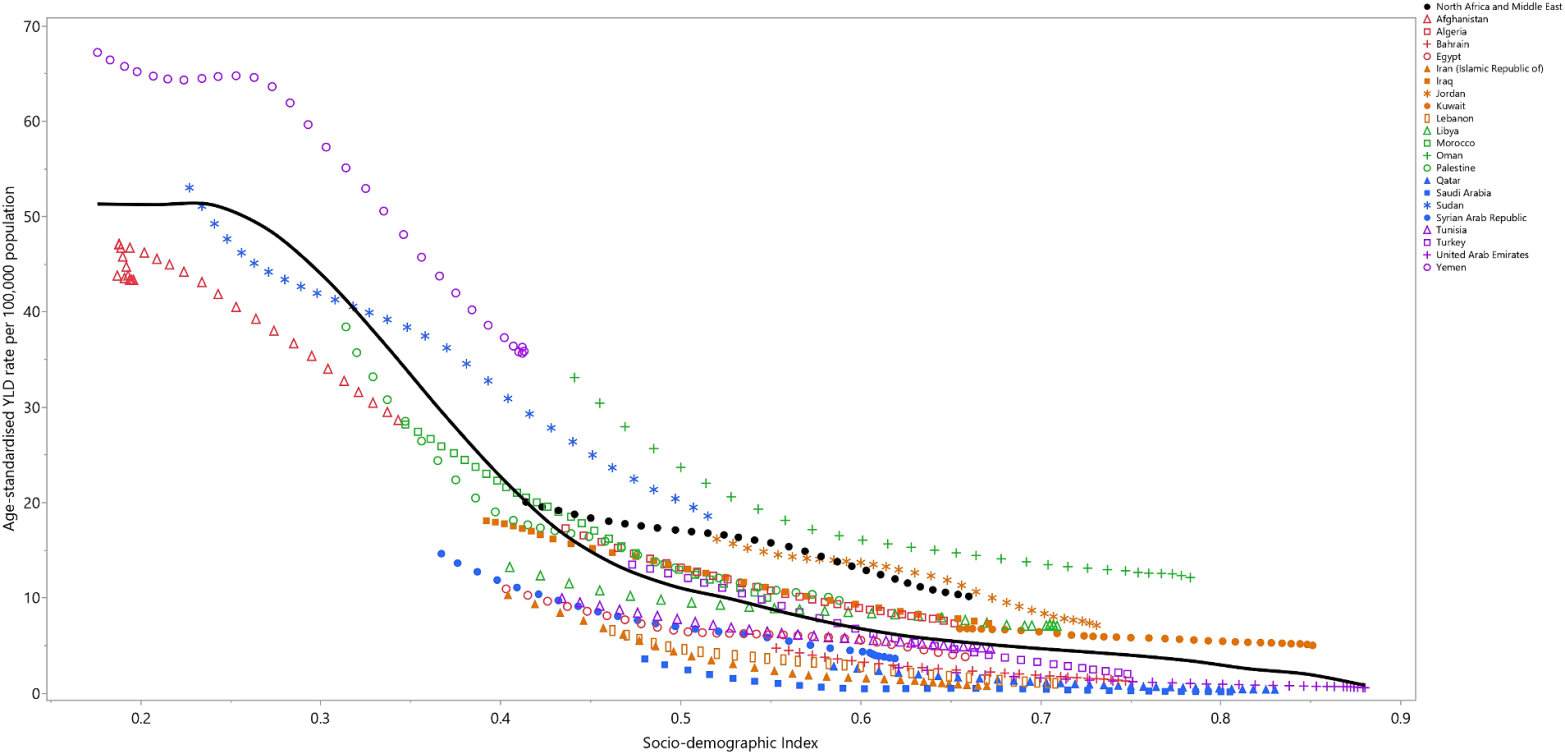
Age-standardized YLD rates of vitamin A deficiency for the 21 MENA countries and territories in 2019, by SDI; Expected values based on the Socio-demographic Index and disease rates in all locations are shown as the black line. Each point shows the observed age-standardized YLD rate for each country in 2019. YLD = years-lived-with-disability. SDI = Socio-demographic Index (Generated from data available from http://ghdx.healthdata.org/gbd-results-tool).

In MENA, data from 1990 to 2019 revealed an inverse relationship between the Socio-demographic Index (SDI) and the YLD rate attributable to VAD (Figure 4). The YLD rate remained stable up to an SDI of 0.25, then dramatically decreased up to an SDI of 0.5, and then steadily declined as the SDI level increased. Throughout the measurement period, Yemen, Oman, and Jordan exhibited larger than expected YLD rates, while Afghanistan, Iran, Egypt, Tunisia, Iran, Lebanon, Bahrain, Saudi Arabia, and the Syrian Arab Republic exhibited smaller than expected rates, based on their SDI levels (Figure 4).

## Discussion

This research represents the first comprehensive documentation of the prevalence and YLDs attributable to VAD across the 21 MENA countries from 1990 to 2019. The MENA region had 30.6 million prevalent cases in 2019, with an age-standardized prevalence rate of 5249.9 per 100,000. In addition, VAD was responsible for 62.2 thousand YLDs, resulting in an age-standardized YLD rate of 10.2 per 100,000. Furthermore, both the age-standardized prevalence and YLD rates of VAD have decreased significantly between 1990 and 2019. Among the MENA nations, Afghanistan, Yemen, and Sudan exhibited the highest age-standardized prevalence and YLD rates, whereas the lowest rates were found in Saudi Arabia, Qatar, and the United Arab Emirates. Furthermore, using the WHO cut-off values for public health significance, our study found that VAD was a severe public health problem in Afghanistan and a moderate public health problem in Sudan. The data also revealed that the prevalence and YLD rates of VAD declined with increasing age, showing no significant differences between sexes. In 2019, the VAD-attributable YLD rates were lower in the MENA region than they were globally for both males and females and in all age groups. Finally, SDI was negatively associated with the age-standardized YLD, indicating that higher development levels corresponded with lower YLD rates.

Although no prior research has specifically examined the burden of VAD in MENA, several global (7, 16) and national (17–23) studies have been published. In 2019, one study reported the prevalence of micronutrient deficiencies and non-essential heavy metal levels in adults living in Saudi Arabia (17). The prevalence rate of VAD was found to be 4.8% in males and 10.7% in females (17). Furthermore, contrary to our findings, that study found that males had a considerably lower prevalence rate of VAD than females (17). In addition, their study also found no significant variations in the prevalence rate of VAD between age groups (17). The differences between their study and ours may be due to the recruitment strategies used. The Saudi study only sampled adult individuals living in urban cities and data collection was suspended for 9 months due to the COVID-19 pandemic, which could have altered participants’ dietary habits (17). Moreover, the study defined VAD as a serum retinol level of less than 30 μg/dL (17), whereas our study followed the WHO’s guidelines, which is a serum retinol level of less than 20 μg/dL. In 2011, a nationwide cross-sectional study was undertaken in Iran to examine the level of micronutrient deficiencies (20). This study reported the prevalence rate of VAD to be 18.3% (16.7–19.9) in children aged 15–23 months and 14.1% (12.7–15.5) in pregnant mothers (20). Furthermore, in rural settings the frequency of VAD was found to be considerably higher in male children than among female children (20). Similarly, in 2013 a national cross-sectional study was undertaken in Palestine that focused on children aged 6–59 months (21). The prevalence of VAD was reported to be 73.1%, with no significant difference between males and females (21). However, it should be noted that the study defined VAD as a serum retinol level of less than 30 g/dL, which may have resulted in an overestimation of the prevalence (21).

Despite a substantial reduction in the prevalence of VAD since 1990, the prevalence rate remains high and has one of the largest burdens of all micronutrient deficiencies (1). Vitamin A plays key physiological roles in vision (24), gene regulation (25), immunity and inflammation (26), epithelial homeostasis (27), and embryogenesis (28), with a lack of vitamin A affecting several systems of the human body. In the presence of VAD, the respiratory system is more susceptible to infection and skin keratinization due to epithelial alterations, spermatogenesis is impaired, and epithelial alternation in the gastrointestinal system can occur (29). Nevertheless, the most noticeable consequence of VAD is normally visual impairment (29). This disorder begins with low-light vision impairment and progresses to hyperkeratinization of the ocular epithelial tissue, known as xerophthalmia, which can result in irreversible blindness (24). VAD was ranked as the eighth most common contributor to the age-standardized prevalence of visual loss in 2017 (2). Furthermore, VAD is a risk factor for several causes of mortality in children, including diarrhea and measles (30, 31), and accounts for about 0.4% of all mortality among those under 5 years old in Central Asia and MENA (31).

The age-standardized YLD rate of VAD attributable vision loss has been found to be negatively associated with the human development index (2), and our study found it was negatively associated with the SDI. The high prevalence of VAD makes it a significant public health issue, particularly among pregnant women and children in under-developed countries (32, 33). Malnutrition is the leading cause of micronutrient deficiencies in resource-limited countries (32, 34). In addition, the vitamin A status of breast milk from women with VAD is poor, resulting in their babies also suffering from VAD (35, 36) and predisposing children to infections, which leads to the further progression of VAD (37, 38). In contrast, in resource-rich countries, micronutrition occurs among individuals with underlying medical disorders or atypical diets (34).

Supplementation, fortification, and dietary variety are the main approaches to overcoming VAD. Vitamin A supplementation is the most commonly utilized method because it is inexpensive, ranging from $0.5 to $1.50 (39–41), and studies have shown it to reduce the burden of VAD. A 2017 Cochrane review of 47 studies found that vitamin A supplementation in those aged 6 months to 5 years old could reduce the risk of all-cause death by 12% compared to the control group (42). In addition, vitamin A supplementation has been found to be effective in reducing the: diarrhea incidence rate and mortality rate, measles incidence rate, and ophthalmic complication incidence rate (42). In populations with endemic VAD, the WHO recommends routine periodic Vitamin A supplementation for children aged 6 months to 5 years old, along with other complementary strategies. Due to the proven efficacy of vitamin A supplementation in reducing the disease burden, the universal vitamin A program has been implemented in over 80 countries (42, 43).

Unlike vitamin A supplementation, the effectiveness of food fortification remains a topic of debate. A 2019 Cochrane review, which analyzed 10 randomized controlled trials, concluded that fortifying staple foods with vitamin A alone had minimal to no effect on serum retinol concentrations. However, the review also found that fortifying staple foods with a combination of vitamin A and other micronutrients yielded some benefits. These included an improvement in serum retinol concentration (mean difference 0.22 mol/L 95% CI 0.15–0.30) and a reduction in the risk of subclinical VAD (relative risk 0.71, 95% CI 0.52–0.98) (44). However, fortifying food in the Middle East has been found to be limited and ineffective (45). Future research is required to examine the effect of food fortification on VAD, as well as establishing policies to encourage governments to fortify foods.

The Eastern Mediterranean Region nutrition transition has shifted children’s diets toward processed, energy-dense, and nutrient-poor foods, replacing their previously diverse and healthy diets (46, 47). The traditional Mediterranean diet, with its emphasis on fruit, vegetables, whole grains, legumes, nuts, and seeds, has gradually been supplanted by the more convenient and heavily promoted Western-style dietary patterns, which contributes to the excessive weight gain while at the same time masking nutritional inadequacies (45, 48). The results of the present study underscore the importance of addressing the nutritional transition. Educational and public health interventions are critical for promoting healthy diets, preventing excessive weight gain, and addressing micronutrient deficiencies in the children of this region.

### Study strengths and limitations

The current study represents the first attempt to measure the burden of VAD across the MENA region, as well as within its constituent nations, spanning the period from 1990 to 2019. Our work acknowledges the previously described methodological limitations of GBD 2019. Furthermore, the GBD 2019 study evaluated the prevalence of VAD using the ST-GPR vitamin A supplementation model to produce appropriate estimates. However, the validity and reliability of our estimations were entirely dependent upon the nonfatal database’s quality and quantity, as well as the limited data on vitamin A supplementation acquired from health surveys. Furthermore, despite the fact that vitamin A serves vital functions in multiple human body systems and is a risk factor for several diseases in children, the computation of YLDs relied entirely on the Disability Weights (DW) associated with vision impairment. As a result, people without vision impairment were classified as asymptomatic, causing the burden to be underestimated. Furthermore, measuring the DWs required assessing the severity of visual loss, which is difficult in youngsters due to language and educational deficiencies, as well as limited night-time mobility. According to the WHO Vitamin and Mineral Nutrition Information System database, “corneal scar” was the case definition of vision loss as a result of VAD (49). However, it is important to note that corneal scars can arise for a variety of reasons, other than VAD, which may result in an overestimation of the burden of VAD. These estimates should be treated with caution, given to the scarcity of epidemiological research on VAD in low-income countries and nations experiencing social instability and conflict, as is the case in Afghanistan, Sudan, and Yemen. Finally, pregnant women are particularly susceptible to vitamin A deficiency, highlighting the need for further research to focus on the prevalence of VAD within this important population group.

## Conclusion

The prevalence of VAD has decreased since 1990 and in MENA it can now be classified as a mild public health issue, based on the WHO cut-off values for public health significance. However, given the negative association between the burden of VAD and the SDI, VAD remains a severe public health concern in Afghanistan and a moderate public health issue in Sudan. Our findings highlight the importance of frequently updating health data to ensure its accuracy and relevance. Furthermore, there is a pressing need for the development and implementation of comprehensive guidelines and regulations aimed at supporting the prevention, early detection, and effective treatment of VAD in the MENA region. This is particularly important for countries such as Afghanistan and Sudan, where the burden of VAD may be more pronounced.

## Author’s note

This study is based on publicly available data and solely reflects the opinion of its authors and not that of the Institute for Health Metrics and Evaluation.

## Data availability statement

The datasets presented in this study can be found in online repositories. The names of the repository/repositories and accession number(s) can be found below: the data used for these analyses are all publicly available at: http://ghdx.healthdata.org/gbd-results-tool.

## Ethics statement

The studies involving humans were approved by the Ethics Committee at the Shahid Beheshti University of Medical Sciences (IR.SBMU.RETECH.REC.1402.045). The studies were conducted in accordance with the local legislation and institutional requirements. The participants provided their written informed consent to participate in this study.

## Author contributions

SS: Conceptualization, Data curation, Formal analysis, Investigation, Methodology, Project administration, Resources, Software, Supervision, Validation, Visualization, Writing – original draft, Writing – review & editing. SM: Writing – original draft, Writing – review & editing. SN: Writing – original draft, Writing – review & editing. KM: Writing – original draft, Writing – review & editing. NK: Writing – original draft, Writing – review & editing. MS: Writing – original draft, Writing – review & editing. A-AK: Writing – original draft, Writing – review & editing. MA: Funding acquisition, Writing – original draft, Writing – review & editing.

## Supplementary material

SUPPLEMENTARY FIGURE S1 The percentage change in the age-standardized point prevalence of vitamin A deficiency in the Middle East and North Africa region from 1990 to 2019, by sex and country. (Generated from data available from http://ghdx.healthdata.org/gbd-results-tool).

SUPPLEMENTARY FIGURE S2 The percentage change in the age-standardized YLD rate of vitamin A deficiency in the Middle East and North Africa region from 1990 to 2019, by sex and country. YLD = years-lived-with-disability. (Generated from data available from http://ghdx.healthdata.org/gbd-results-tool).
