# Supplementary figures and images for: Vitamin A deficiency in the MENA region: a 30-year analysis (1990–2019)
Source: Front Nutr. 2024 Jun 6;11:1413617. doi: 10.3389/fnut.2024.1413617 (PMC11187328; doi:10.3389/fnut.2024.1413617)

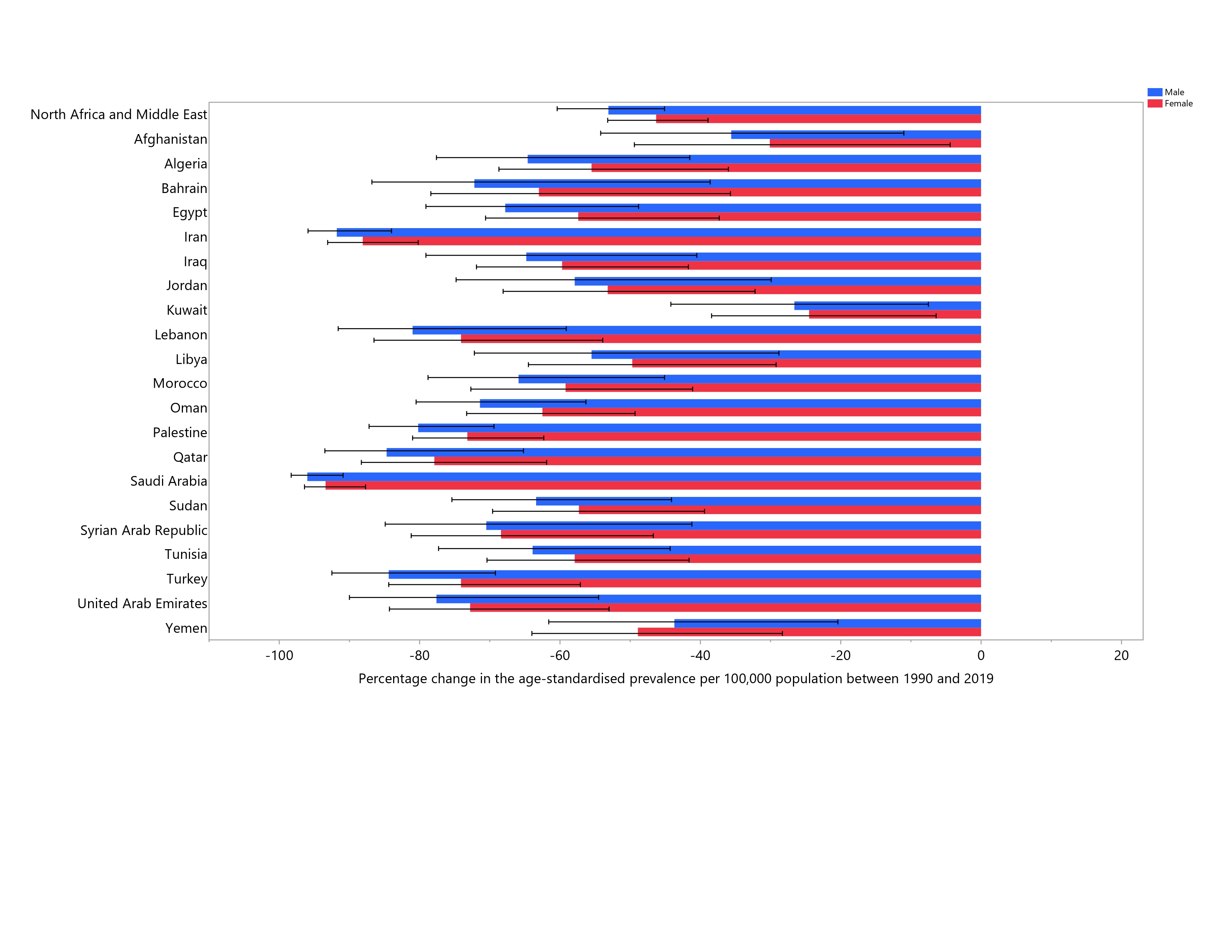

Supplement: SUPPLEMENTARY FIGURE S1 — The percentage change in the age-standardized point prevalence of vitamin A deficiency in the Middle East and North Africa region from 1990 to 2019, by sex and country. (Generated from data available from http://ghdx.healthdata.org/gbd-results-tool). [file Image_1.JPEG]

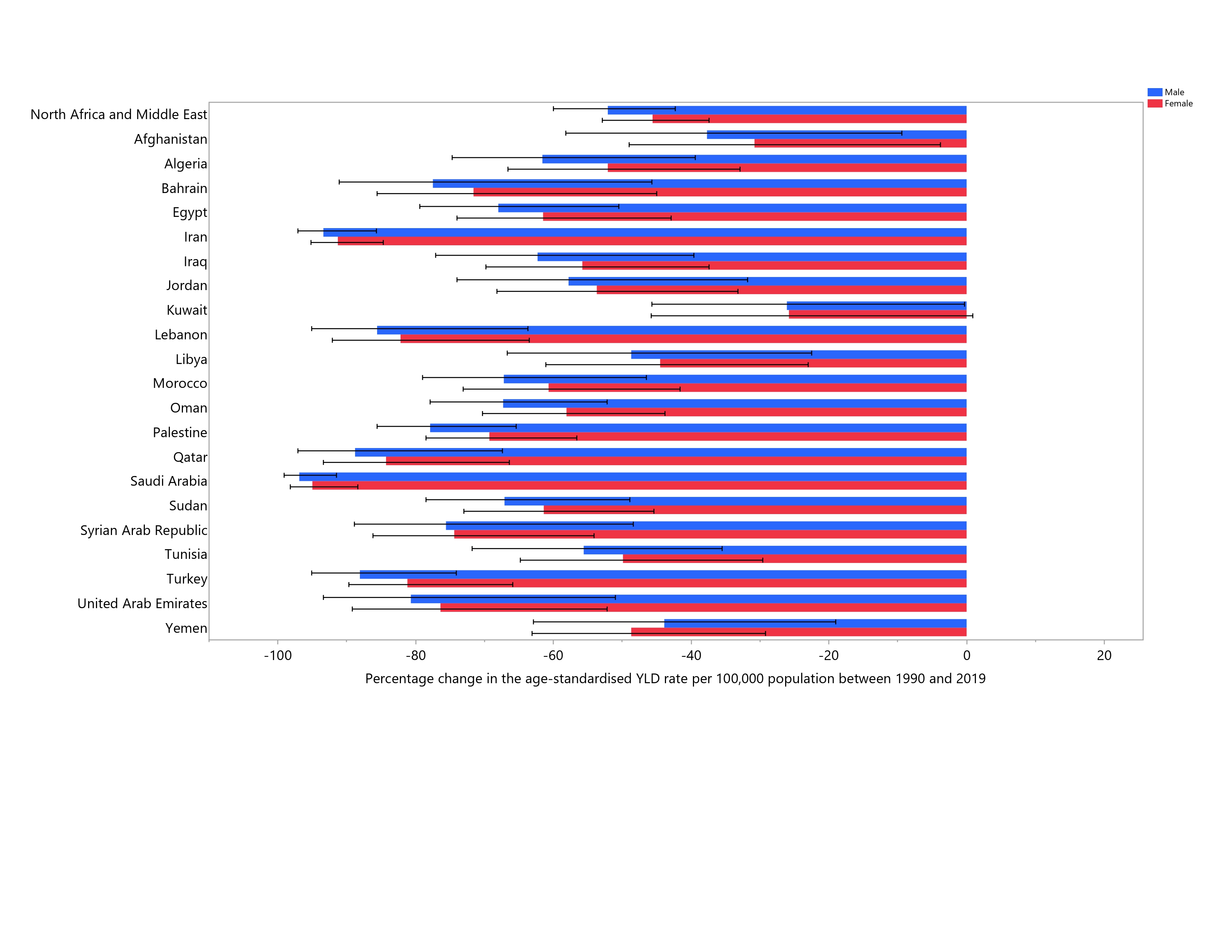

Supplement: SUPPLEMENTARY FIGURE S2 — The percentage change in the age-standardized YLD rate of vitamin A deficiency in the Middle East and North Africa region from 1990 to 2019, by sex and country. YLD = years-lived-with-disability. (Generated from data available from http://ghdx.healthdata.org/gbd-results-tool). [file Image_2.JPEG]
