# Supplementary material for: Vitamin A deficiency in the MENA region: a 30-year analysis (1990–2019)
Source: Front Nutr. 2024 Jun 6;11:1413617. doi: 10.3389/fnut.2024.1413617 (PMC11187328; doi:10.3389/fnut.2024.1413617)
Supplement: Supplementary file 3 [file Table_1.DOCX]

| **Table S1: The severity levels, lay descriptions and disability weights (DWs) of vitamin A deficiency in the Global Burden of Disease Study 2019** | | | |
| --- | --- | --- | --- |
| **Sequela** | **Health state**  **name** | **Lay description** | **Disability weight**  **(95% CI)** |
| Moderate vision  Impairment/loss due to vitamin A deficiency | Distance vision,  Moderate impairment | has vision problems that make it difficult to recognise faces or objects across a room. | 0.031  (0.019–0.049) |
| Severe vision  Impairment/loss due to vitamin A deficiency | Distance vision,  Severe impairment | has severe vision loss, which causes difficulty in daily activities, some emotional impact (for example worry), and some difficulty going outside the home without assistance. | 0.184  (0.125–0.258) |
| Distance vision blindness | Severe | is completely blind, which causes great difficulty in some daily activities, worry and anxiety, and great difficulty going outside the home without assistance. | 0.187  (0.124–0.26) |
| Asymptomatic | - | - | - |
| CI: Confidence interval | | | |
