# Supplementary material for: Vitamin A deficiency in the MENA region: a 30-year analysis (1990–2019)
Source: Front Nutr. 2024 Jun 6;11:1413617. doi: 10.3389/fnut.2024.1413617 (PMC11187328; doi:10.3389/fnut.2024.1413617)
Supplement: Supplementary file 4 [file Table_2.DOCX]

| **Table S2: Prevalence of vitamin A deficiency in 1990 and 2019 and the percentage change in the age-standardised rates (ASRs) per 100,000 in the Middle East and North Africa region**  **(Generated from data available from http://ghdx.healthdata.org/gbd-results-tool)** | | | | | |
| --- | --- | --- | --- | --- | --- |
|  | **1990** | | **2019** | | **Percentage change in ASRs per 100,000** |
|  | **No (95% UI)** | **ASRs per 100,000 (95% UI)** | **No (95% UI)** | **ASRs per 100,000 (95% UI)** |  |
| **North Africa and Middle East** | **53157209 (51155460 , 55198551)** | **15427.7 (14749.2 , 16089.9)** | **30635707 (28841772 , 32427862)** | **5249.9 (4905.9 , 5602.5)** | **-50.3 (-55.9 , -44.7)** |
| **Afghanistan** | **4775285 (4362398 , 5217509)** | **41738.5 (37517.6 , 46253.4)** | **9976079 (8681044 , 11360871)** | **26013.8 (22250.9 , 30088.7)** | **-33.2 (-47.1 , -15)** |
| **Algeria** | **2732045 (2269730 , 3238700)** | **10831.3 (8778.7 , 13077.8)** | **1104506 (936742 , 1321371)** | **2690.4 (2223.7 , 3276.3)** | **-60.9 (-70.6 , -44.6)** |
| **Bahrain** | **24526 (20336 , 29684)** | **4847 (3969.5 , 5928)** | **20747 (17263 , 24956)** | **1459.9 (1201 , 1773.1)** | **-67.9 (-79.4 , -47.1)** |
| **Egypt** | **4262829 (3690659 , 4964002)** | **7674.9 (6504.2 , 9108.4)** | **1909494 (1600082 , 2270531)** | **1925.2 (1590.4 , 2320.4)** | **-62.9 (-72.7 , -50)** |
| **Iran (Islamic Republic of)** | **6380161 (5501497 , 7379382)** | **11013.2 (9319.2 , 12973.8)** | **1105547 (942030 , 1334897)** | **1344.7 (1118.9 , 1651.8)** | **-90.4 (-93.9 , -84.7)** |
| **Iraq** | **2498648 (2126113 , 2909933)** | **14157.6 (11755.1 , 16890)** | **1337629 (1114027 , 1602377)** | **3209.2 (2619.1 , 3937.9)** | **-62.7 (-73.6 , -47.4)** |
| **Jordan** | **537722 (458785 , 622744)** | **14577.8 (12277.1 , 17103.8)** | **575229 (487422 , 683785)** | **5045.5 (4187.4 , 6131.2)** | **-55.8 (-68.1 , -39)** |
| **Kuwait** | **38964 (31996 , 46832)** | **2226.3 (1809.9 , 2713.2)** | **32538 (27566 , 39139)** | **745.7 (619.9 , 912.2)** | **-25.6 (-38.7 , -12.5)** |
| **Lebanon** | **229237 (194023 , 270877)** | **7051.5 (5831.5 , 8495.5)** | **85320 (72497 , 101034)** | **1682.7 (1399.5 , 2043.8)** | **-77.9 (-86.7 , -63.3)** |
| **Libya** | **325729 (269666 , 384398)** | **7697 (6236.3 , 9246.1)** | **121053 (101641 , 144469)** | **1829.2 (1515.3 , 2213.5)** | **-52.8 (-66.1 , -36.3)** |
| **Morocco** | **5074281 (4390583 , 5824558)** | **20349.7 (17273.5 , 23794)** | **2140590 (1798005 , 2534391)** | **6117.3 (5055.5 , 7318.7)** | **-63.2 (-73.2 , -48.8)** |
| **Oman** | **259460 (216358 , 308661)** | **13315.5 (10911.8 , 16069.3)** | **59235 (48447 , 74110)** | **1300.8 (1050.1 , 1633.5)** | **-67.6 (-75.7 , -57.1)** |
| **Palestine** | **876747 (788829 , 962853)** | **42555.1 (37960.6 , 47276.6)** | **266716 (222099 , 324426)** | **5435.6 (4444.9 , 6784.1)** | **-77.7 (-83.4 , -69.4)** |
| **Qatar** | **16332 (13184 , 20161)** | **3681.3 (2955.7 , 4576.5)** | **20586 (16451 , 26077)** | **725.8 (575.1 , 927.4)** | **-81.5 (-89.1 , -70.5)** |
| **Saudi Arabia** | **630814 (513241 , 796583)** | **3938.5 (3124 , 5166.3)** | **66898 (56035 , 78991)** | **190 (156.5 , 229)** | **-95 (-97.2 , -91.2)** |
| **Sudan** | **7410191 (6682407 , 8221310)** | **36394.4 (32214.5 , 40852.6)** | **3681595 (3088138 , 4399984)** | **9067.5 (7431.2 , 11071.5)** | **-61.1 (-70.2 , -48.6)** |
| **Syrian Arab Republic** | **1830518 (1526769 , 2163998)** | **14119.5 (11651.7 , 17057.5)** | **529821 (446326 , 624305)** | **3722.4 (3067.3 , 4442.2)** | **-69.6 (-80.5 , -53.5)** |
| **Tunisia** | **691739 (578212 , 820714)** | **8202.7 (6753.4 , 10020.2)** | **202042 (173828 , 233624)** | **1755 (1468 , 2071.5)** | **-61.3 (-71.8 , -47.9)** |
| **Turkey** | **8301116 (7435587 , 9192889)** | **13891.2 (12333.7 , 15509.1)** | **2574870 (2218998 , 2951848)** | **3184.3 (2713.4 , 3724.8)** | **-79.9 (-86.8 , -69.8)** |
| **United Arab Emirates** | **60346 (49595 , 72877)** | **3233.2 (2620.1 , 3937.2)** | **66510 (52584 , 84088)** | **729.8 (578.1 , 919.2)** | **-75 (-84.4 , -61.2)** |
| **Yemen** | **6164768 (5622496 , 6737433)** | **44607.6 (40223.6 , 49336.3)** | **4727579 (3968589 , 5544227)** | **15059.3 (12423.1 , 17887.4)** | **-46 (-58.9 , -30)** |
