# Supplementary material for: Vitamin A deficiency in the MENA region: a 30-year analysis (1990–2019)
Source: Front Nutr. 2024 Jun 6;11:1413617. doi: 10.3389/fnut.2024.1413617 (PMC11187328; doi:10.3389/fnut.2024.1413617)
Supplement: Supplementary file 5 [file Table_3.DOCX]

| **Table S3: YLDs due to vitamin A deficiency in 1990 and 2019 and the percentage change in the age-standardised rates (ASRs) per 100,000 in the Middle East and North Africa region**  **(Generated from data available from http://ghdx.healthdata.org/gbd-results-tool)** | | | | | |
| --- | --- | --- | --- | --- | --- |
|  | **1990** | | **2019** | | **Percentage change in ASRs per 100,000** |
|  | **No (95% UI)** | **ASRs per 100,000 (95% UI)** | **No (95% UI)** | **ASRs per 100,000 (95% UI)** |  |
| **North Africa and Middle East** | **101246 (68222 , 145753)** | **20.1 (13.5 , 28.8)** | **62161 (41276 , 87376)** | **10.2 (6.7 , 14.3)** | **-49.3 (-55.3 , -43.1)** |
| **Afghanistan** | **7878 (5034 , 11557)** | **43.8 (27.8 , 64.2)** | **17489 (10887 , 25833)** | **28.6 (18 , 42.3)** | **-34.6 (-49.4 , -14.1)** |
| **Algeria** | **6063 (3639 , 9158)** | **17.3 (10.5 , 25.9)** | **3078 (1917 , 4712)** | **7.3 (4.6 , 11.2)** | **-57.6 (-68.7 , -41.7)** |
| **Bahrain** | **28 (16 , 47)** | **4.7 (2.7 , 8)** | **9 (5 , 17)** | **1.2 (0.6 , 2.1)** | **-74.7 (-85.5 , -55.7)** |
| **Egypt** | **8384 (5232 , 12641)** | **10.9 (6.8 , 16.6)** | **4196 (2572 , 6518)** | **3.8 (2.4 , 6)** | **-65 (-74.7 , -52.9)** |
| **Iran (Islamic Republic of)** | **9549 (5821 , 14855)** | **10.3 (6.3 , 16)** | **540 (284 , 986)** | **0.8 (0.4 , 1.4)** | **-92.7 (-95.7 , -87.3)** |
| **Iraq** | **4949 (3054 , 7595)** | **18.1 (11.3 , 27.2)** | **3353 (2050 , 5099)** | **7.3 (4.5 , 11.1)** | **-59.6 (-71.3 , -45.3)** |
| **Jordan** | **885 (550 , 1334)** | **16.2 (10.1 , 24.1)** | **882 (538 , 1335)** | **7.1 (4.4 , 10.7)** | **-55.9 (-68.5 , -38.1)** |
| **Kuwait** | **130 (82 , 195)** | **6.8 (4.3 , 10.1)** | **197 (117 , 305)** | **5 (3.1 , 7.7)** | **-26 (-41.1 , -9.2)** |
| **Lebanon** | **297 (161 , 488)** | **6.6 (3.6 , 10.9)** | **52 (25 , 102)** | **1 (0.5 , 2.1)** | **-84.1 (-91.7 , -70.9)** |
| **Libya** | **791 (498 , 1215)** | **13.2 (8.5 , 19.9)** | **418 (261 , 627)** | **7 (4.4 , 10.4)** | **-46.8 (-60.3 , -30.7)** |
| **Morocco** | **9535 (5879 , 14628)** | **28.2 (17.4 , 43.2)** | **3353 (1997 , 4962)** | **10 (5.9 , 14.9)** | **-64.5 (-74.3 , -52)** |
| **Oman** | **954 (616 , 1451)** | **33.1 (21.5 , 50)** | **532 (333 , 788)** | **12.1 (7.7 , 18)** | **-63.3 (-73.1 , -52.8)** |
| **Palestine** | **1334 (815 , 2019)** | **38.4 (23.2 , 58.1)** | **582 (357 , 876)** | **9.7 (6 , 14.5)** | **-74.7 (-81.5 , -65.2)** |
| **Qatar** | **13 (7 , 21)** | **2.8 (1.5 , 4.8)** | **6 (3 , 11)** | **0.4 (0.2 , 0.7)** | **-86.5 (-93.2 , -74.6)** |
| **Saudi Arabia** | **884 (471 , 1564)** | **3.6 (1.9 , 6.4)** | **32 (14 , 63)** | **0.1 (0.1 , 0.3)** | **-96.2 (-98.3 , -92.4)** |
| **Sudan** | **17337 (10909 , 25166)** | **53 (33.6 , 77.2)** | **9776 (5951 , 14693)** | **18.6 (11.3 , 27.8)** | **-64.9 (-73.6 , -53.3)** |
| **Syrian Arab Republic** | **3226 (1874 , 5385)** | **14.6 (8.5 , 24.7)** | **460 (237 , 808)** | **3.6 (1.9 , 6.5)** | **-75.1 (-84.9 , -58.3)** |
| **Tunisia** | **1040 (661 , 1584)** | **9.9 (6.4 , 14.8)** | **490 (305 , 744)** | **4.7 (2.9 , 7.1)** | **-53.1 (-65.6 , -39)** |
| **Turkey** | **10219 (6263 , 16111)** | **13.5 (8.2 , 21.3)** | **1060 (541 , 1860)** | **2 (1 , 3.5)** | **-85 (-91.2 , -75.6)** |
| **United Arab Emirates** | **57 (30 , 99)** | **2.7 (1.4 , 4.5)** | **23 (11 , 41)** | **0.6 (0.3 , 1)** | **-78.4 (-88.1 , -61.9)** |
| **Yemen** | **17625 (11850 , 25279)** | **67.2 (45 , 96.5)** | **15569 (10107 , 23336)** | **36.3 (23.7 , 53.6)** | **-46 (-58.7 , -29.8)** |
